# The Importance of the Lifelike Esthetic Appearance of All-Ceramic Restorations on Anterior Teeth

**DOI:** 10.1155/2015/704348

**Published:** 2015-01-29

**Authors:** Daniela Micheline dos Santos, Amália Moreno, Aljomar José Vechiato-Filho, Liliane da Rocha Bonatto, Aldiéris Alves Pesqueira, Murilo César Bento Laurindo Júnior, Rodrigo Antonio de Medeiros, Emily Vivianne Freitas da Silva, Marcelo Coelho Goiato

**Affiliations:** Department of Dental Materials and Prosthodontics, Aracatuba Dental School, Universidade Estadual Paulista (UNESP), 16015050 Aracatuba, SP, Brazil

## Abstract

The success of rehabilitation will not depend on just clinical procedures. A proper dental technique (ceramist) is required as well as the respect for some biomimetic principles to obtain the desired final result. This study has the purpose of describing a prosthetic rehabilitation with laminate veneers and all-ceramic crowns of a patient unsatisfied with a previous esthetic treatment because of the negligence of some biomimetic principles. A 45-year-old female patient was admitted to the dental clinic complaining about the lifelike appearance of her all-ceramic restorations. Before the fabrication of new restorations, a mock-up was conducted to verify the patient's satisfaction. A ceramist conducted all the fabrication process so that surface characterizations could be visually verified and the lifelike appearance of natural tooth could be reproduced. After the cementation procedure, the patient reported being satisfied with the lifelike appearance of the new restorations. Based on the clinical findings of the present case report, it can be concluded that the reproduction of the lifelike esthetic appearance of natural teeth and the visualization of the final results before definitive procedures are essential to obtain the clinical success.

## 1. Introduction

Since their introduction on clinical routine, the porcelain laminate veneers and all-ceramic crowns have proven to have satisfactory long-term esthetic results superior to other restorative materials as long as they are properly planned and fabricated [1–9]. This fact increased the popularity of all-ceramics restorations and led to a progressive demand of the patients for a high level of esthetic treatments. Besides, published studies report other advantages of this new restorative modality, that is, the integration of the restoration with the surrounding periodontal tissue and thus the patient's self-steam [2, 4, 10].

To date, there are several ceramic systems available on dentistry market that may reproduce the teeth with great naturally [2]. However, the patient's esthetic desires depend on the adequate application of techniques. If some clinical details are not observed and carefully analyzed, the final result might be compromised and the rehabilitation led to failure [1, 11]. Some strategies may be used to prevent those complications such as the mock-up technique [12], clinician and laboratory technical communication, and the association of photographs with digital programs that will provide to both patient and dentist [2, 13] a 3-dimensional visualization of the final result, that is, Adobe Photoshop Smile Design (PSD) [14] or DSD (Digital Smile Design).

Nevertheless, the success of rehabilitation will not depend on just clinical procedures. A proper dental technique (ceramist) is required and should have special skills [3, 15] and respect some biomimetic principles to obtain the desired final result, such as personal characterizations (stains), the level of enamel translucence, and its surface texture [13]. Based on the relevance of esthetic treatments with all-ceramic restorations on clinical routine, this study has the purpose of describing a prosthetic rehabilitation with laminate veneers and all-ceramic crowns of a patient unsatisfied with a previous esthetic treatment because of the negligence of some biomimetic principles.

## 2. Patient Case Report

A 45-year-old female patient was admitted to the dental clinic (Aracatuba Dental School, Aracatuba, Sao Paulo, Brazil) reporting dissatisfaction to a previous treatment and complaining about the lifelike esthetic appearance of her anterior maxillary teeth, in addition to the grayish discoloration of the gingival margin adjacent to the left central incisor (number 21).

**Figure 1:**
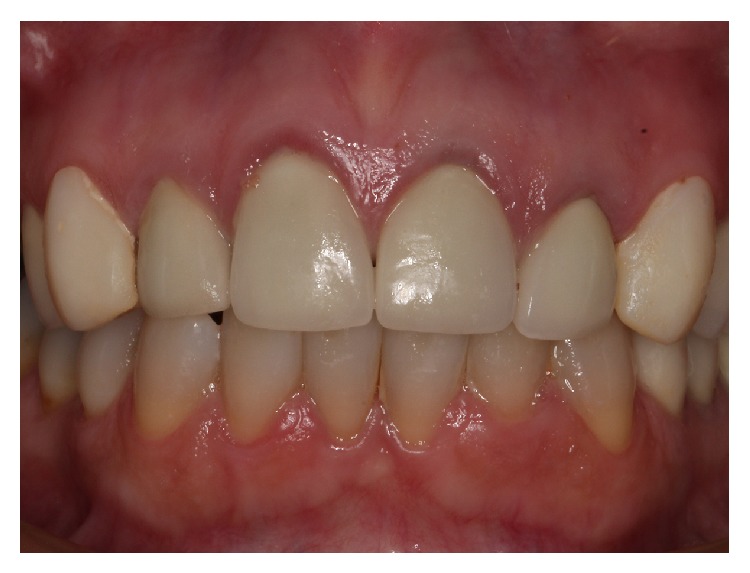
Initial clinical aspect, frontal view.

The intraoral examination revealed the presence of all-ceramic crowns on the anterior maxillary incisors (from number 12 to number 22) and porcelain laminate veneers on canines of the same arch (number 13 and number 23). All those elements showed unacceptable lifelike esthetic appearance, high opacity (mainly on canines), color mismatch, and lack of texture (Figure 1). The contour of the concave arches was disproportionate, leading to gingival discrepancy and malalignment of the central incisors (number 11 and number 21). Besides, the left lateral incisor (number 22) showed a nonproper incisal display, contradicting the principles of a harmonious smile line.

**Figure 2:**
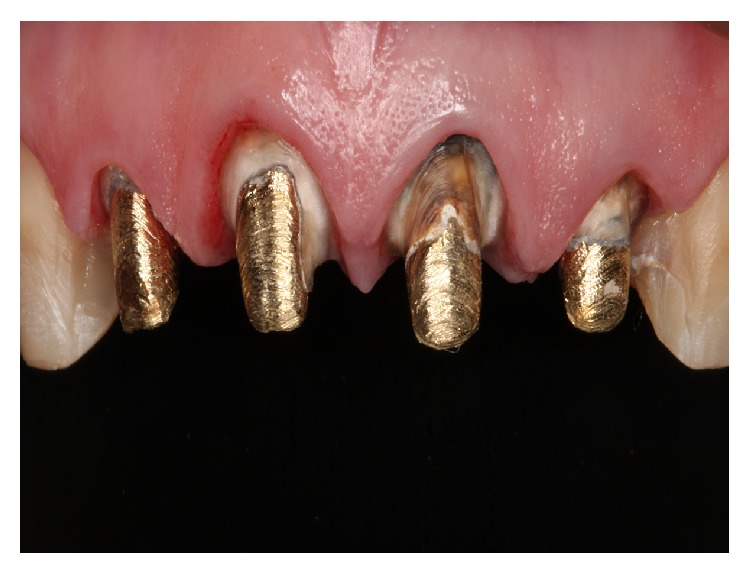
Prepared teeth.

It was decided to replace the all-ceramic restorations through discussion of treatment possibilities. After anamnesis and intraoral exam, diagnostic impressions were performed with irreversible hydrocolloid (Hydrogum, Zhermack SpA Rovigo, Italy); then the casts were mounted in a semiadjustable articulator. The all-ceramic restorations were removed with a diamond bur (number 4138, Kg Sorensen, Cotia, Sao Paulo, Brazil). It was observed that the metal cores of the anterior maxillary incisors were properly adapted and all the restored teeth had a satisfactory preparation design (Figure 2). The diagnostic mock-up was performed with autopolymerizing acrylic resin to obtain a predictable result and patient's full approval (Protemp, 3M, St. Paul, Minnesota, USA). The provisional restorations were fabricated with stock teeth (Trilux, VIPI, Pirassununga, Sao Paulo, Brazil) and autopolymerizing acrylic resin (Classico, Sao Paulo, Sao Paulo, Brazil). The latter were cemented with noneugenol provisional cement (Temrex Temporary Cement, Temrex, New York, USA).

**Figure 3:**
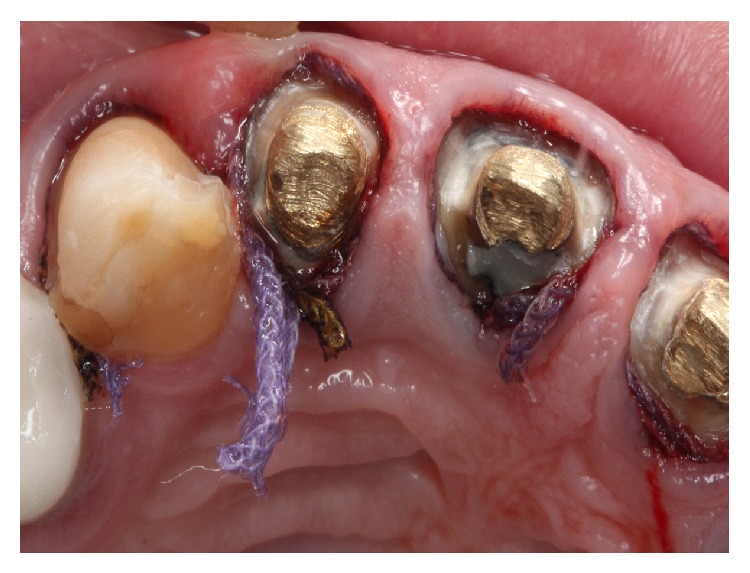
Gingival displacement with retraction cord. Note patient's gingival biotype proper for this impression technique.

**Figure 4:**
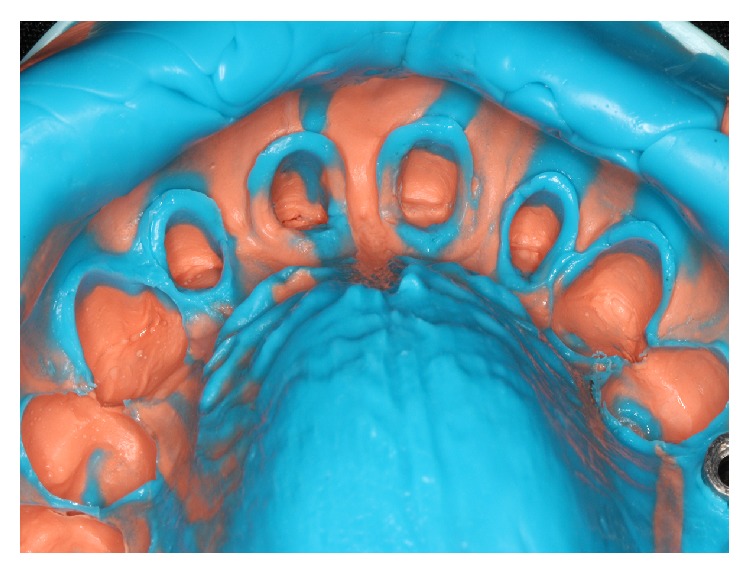
Definitive impression.

**Figure 5:**
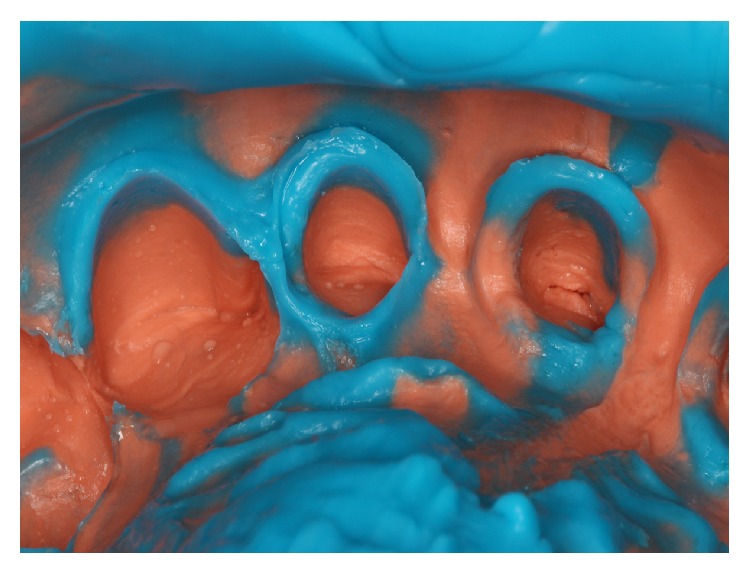
Definitive impression, close-up view of preparations finish line.

The soft tissue displacement was performed with gingival retraction cords (Ultrapak, Ultradent Products Inc., South Jordan, Utah, USA) because the patient gingival biotype showed a satisfactory thickness (Figure 3), minimizing the risks of irreversible gingival recessions. The definitive impression was performed with polysiloxane impression material (3M, St. Paul, Minnesota, USA) (Figures 4 and 5).

A special dental stone type IV (Gesso-Rio, Orlando Antonio Bussioli-ME, Rio Claro, Sao Paulo, Brazil) was poured on the definitive impressions and then conducted to the dental laboratory phase which was performed carefully to avoid the same mistakes observed on the previous all-ceramic restorations. Lithium disilicate ceramic ingots (IPS E.max Press, Ivoclar Vivadent AG, Schaan, Liechtenstein) on medium opacity shade (MO 0) were used to fabricate the laminate veneer coppings and high opacity ingots (HO 0) to all-ceramic crowns. Those ingots are necessary to mask the dark subsurface of the teeth with metal cores and the grayish appearance observed in the right central incisor finish line (number 11). A lithium disilicate layering ceramic was used on A2 color shade for restorations veneering; then their characterization was performed with A2, white and pink (interproximal region) stains, respecting the color nuances necessary to reproduce a lifelike esthetic appearance.

**Figure 6:**
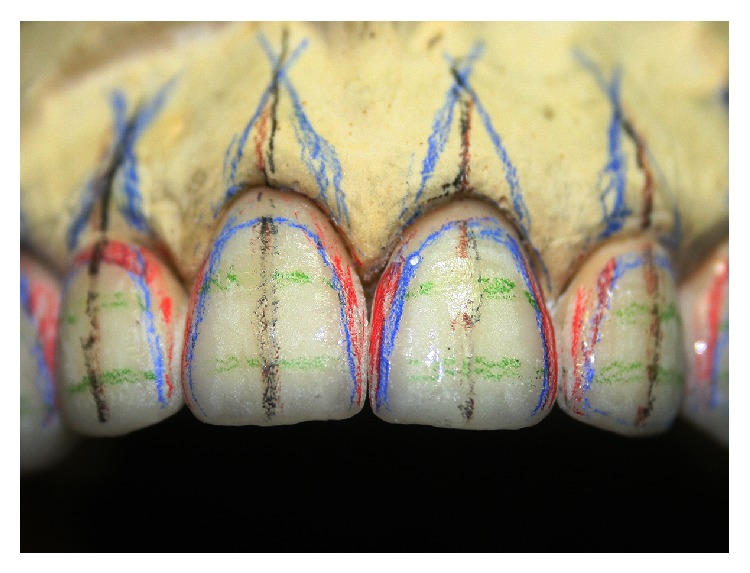
Emergency profile guidelines.

**Figure 7:**
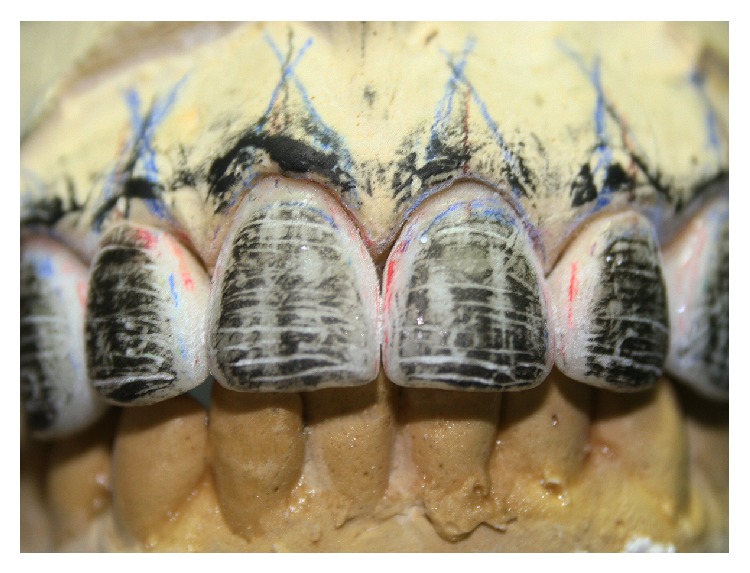
Surface texture reproduction with graphite.

**Figure 8:**
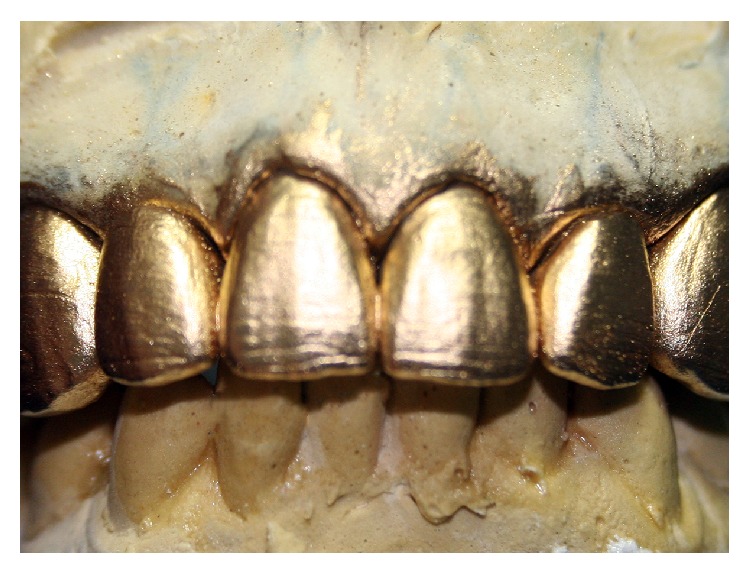
Surface texture reproduction with gold powder.

The restorations were divided into thirds associated with emergency profile guidelines allowing the gradual ceramic layering and whitening the restoration from cervical to incisal plane (Figure 6). Graphite was scratched on the restorations surface to reproduce the lifelike texture and characteristics of natural teeth (Figure 7) and gold powder was applied to visualize the regions of high and low brightness (Figure 8).

**Figure 9:**
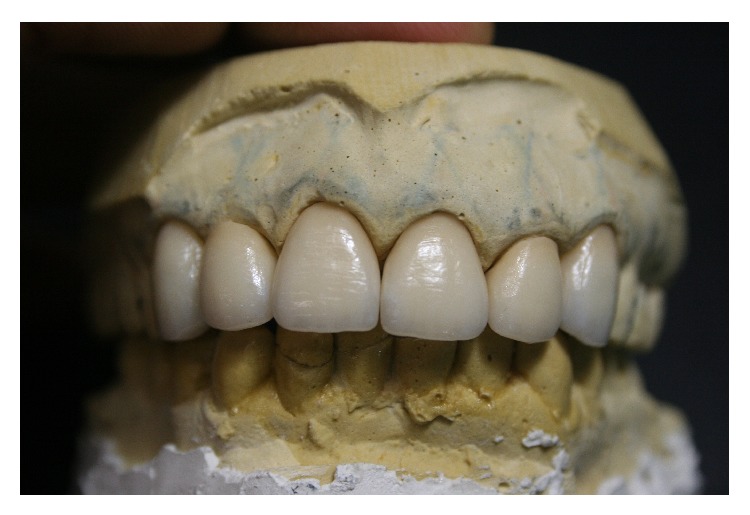
Porcelain laminate veneer.

**Figure 10:**
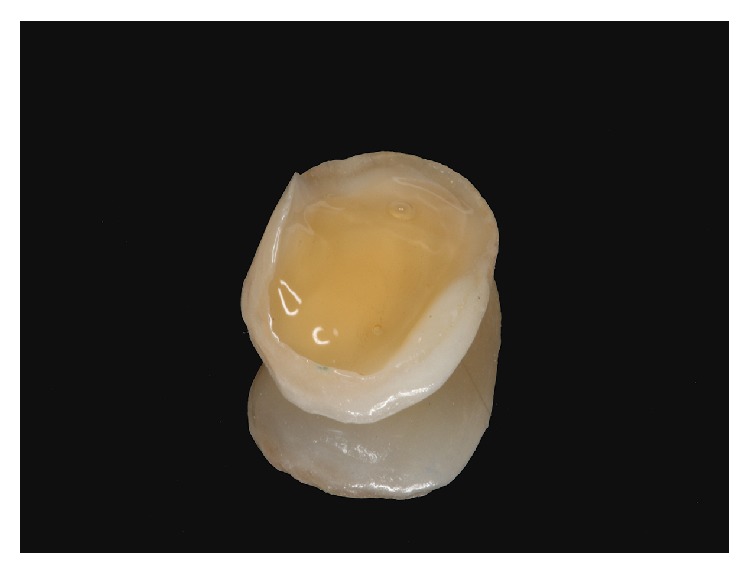
Surface etching with 10% hydrofluoric acid gel.

**Figure 11:**
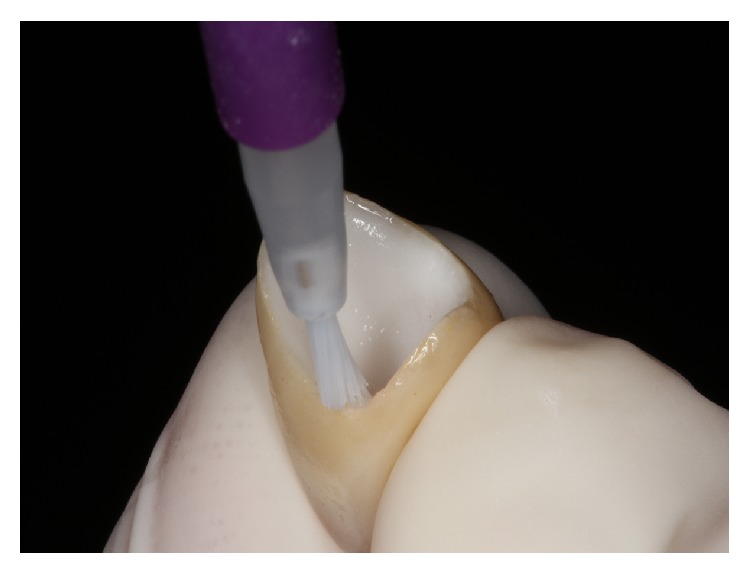
Silane application.

**Figure 12:**
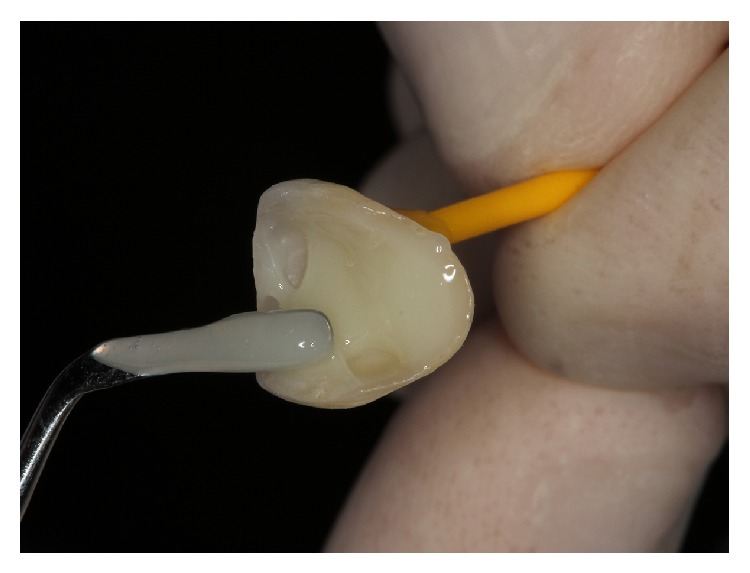
Dual-curing resin cement placement on the inner surface of all-ceramic restoration.

After the all-ceramic restorations were finalized (Figure 9), their inner surface was etched with 10% hydrofluoride acid gel (Dentsply, Petropolis, Rio de Janeiro, Brazil) for 20 seconds according to the manufacturer's instructions (Figure 10). The restorations were rinsed with water spray for 1 minute and then air-dried. Next, silane was applied on the etched surface for 60 seconds (Figure 11); then its excess was removed by air jets, so the restorations were prepared for cementation step. Simultaneously, the prepared teeth were cleaned and etched for 30 seconds with 37% phosphoric acid (Dentsply, Petropolis, Rio de Janeiro, Brazil), rinsed, and dried. The adhesive agent (Monobond Plus, Ivovlar Vivadent AG, Schaan, Liechtenstein) was applied to the surfaces and each layer was photopolymerized for 20 seconds (Figure 12).

**Figure 13:**
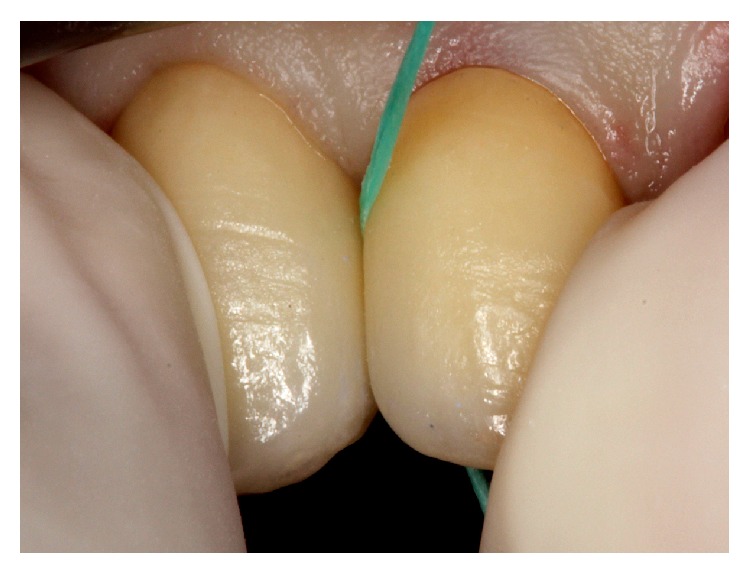
Removal of resin cement excesses.

Based on the try-in, a colorless luting agent was chosen. A transparent dual-curing luting agent (Variolink II, Ivoclar Vivadent AG, Schaan, Liechtenstein) was mixed and applied to the inner surface of the restorations. Then they were placed on the prepared teeth and held in place. Their excesses were removed (Figure 13) and the photopolymerization was performed. The cement mixing and its photopolymerization were conducted according to the manufacturer's instructions.

**Figure 14:**
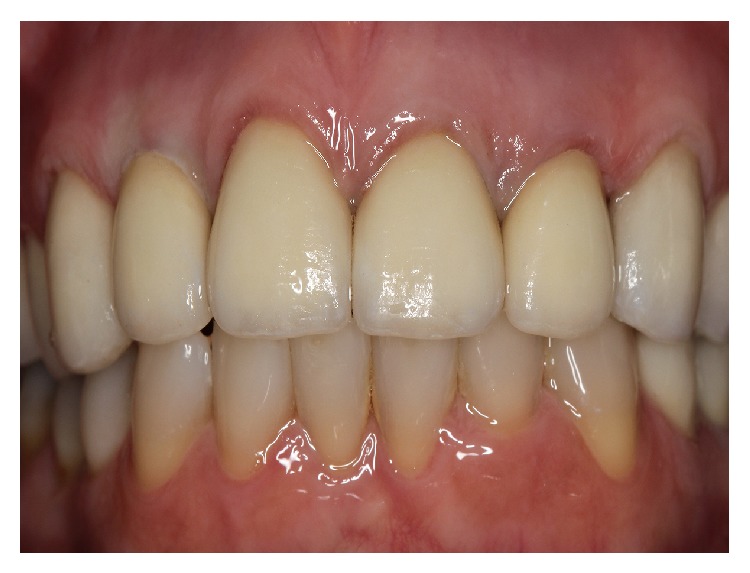
Final aspect of treatment, immediately after cementation procedure.

**Figure 15:**
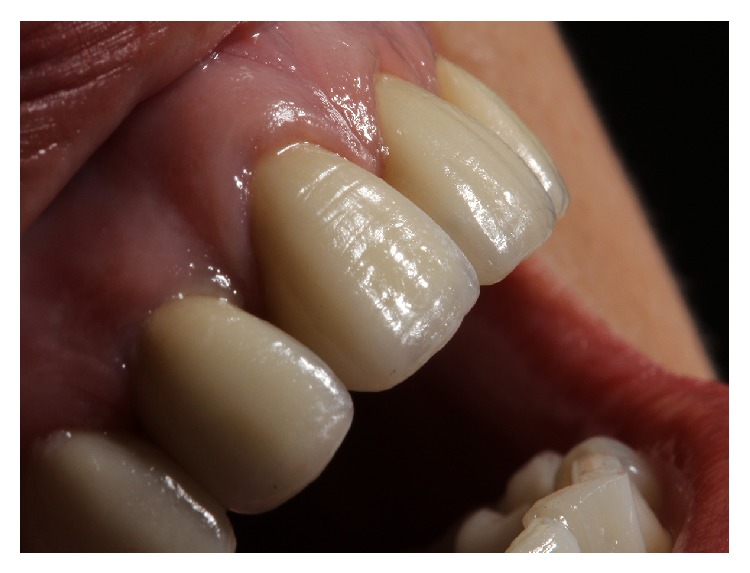
Lifelike esthetic result. Note the light transmission on restorations surface.

**Figure 16:**
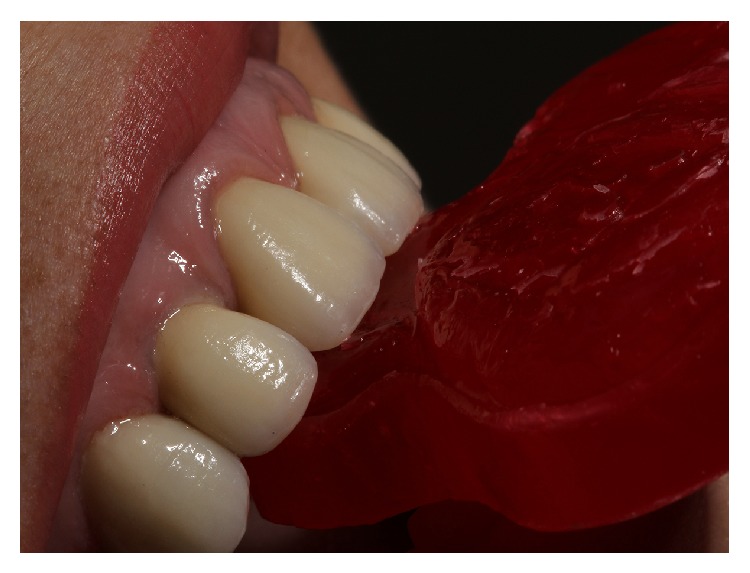
Restorations with lifelike esthetic result.

After the cementation, the color of the all-ceramic restorations matched with the patient's natural teeth (Figure 14). Besides, the dimension of the right central incisor was proportional to the left adjacent tooth and the left lateral incisor was properly located on the smile contour. The concave arches were more harmonic and the grayish discoloration on the gingival margin of the left central incisor (number 22) was minimized. The color graduation and teeth translucence in the incisal third were more detailed and evident (Figures 15 and 16) which reproduced the lifelike esthetic appearance of natural teeth. Those factors contributed to the patient's satisfaction with the esthetic of the restorations and their functional results.

## 3. Discussion

Nowadays, a progressive demand of patients for a high level of esthetic treatments is observed on clinical routine [12]. In the present study, it was verified that despite the fact that all-ceramic restorations may reproduce the lifelike esthetic appearance of natural teeth [3, 16], it depends on an adequate application of techniques such as the reproduction of the enamel surface texture, tooth color, natural characteristics (i.e., stains), tooth morphology, and integration of the restoration with the surrounding periodontal tissue [4, 12].

Despite the previous esthetic treatment with all-ceramic restorations, the patient was unsatisfied with the lifelike esthetic result obtained. One of the reasons that led to patient's dissatisfaction can be observed on Figure 1. According to Chiche and Pinault (1996) [11], the position of the incisors is an important parameter to obtain a harmonic and esthetic smile. However, it can be observed initially that the tooth number 22 showed a nonproper incisal display. This arrangement is contrary to the biomimetic parameters listed above and caused an asymmetry on the smile [2].

To avoid those clinical complications, the literature recommends that the final results are necessary to be visualized before a definitive procedure [11]. An alternative to obtain predictability on the prosthetic treatment is the diagnostic wax-up, the mock-up technique and the clinician and laboratory technical communication for the adequate reproduction of natural tooth characteristics [2, 12, 13].

Nevertheless, the success of the prosthetic treatment depends on the adequate application of laboratory techniques [2, 16]. In the present study, the technician (ceramist) used strategies to verify if the characteristics of natural teeth (Figures 6, 7, and 8). Clinically, it can be observed that those parameters were well performed on Figure 16 based on the interaction of the light to the prostheses surface. The surface texture and morphology of natural teeth affect the quality and quantity of light transmitted to human eyes modifying the color perceptions [17]. Thus, the reproduction of those natural imperfections (biomimetic) was an important factor for a lifelike esthetic result.

Besides, the selection of the cement type and color as well as the restoration placement is essential to obtain clinical success [18]. In the present study, a dual-curing luting agent was used because of the high opacity of the coppings. It may affect the light transmission and the photopolymerization might not be efficient at the deeper parts of the root canal. Thus, the catalyst paste of dual-curing resin cements improves the degree of conversion of the polymerization process [2].

Within the limitations of this study, it can be concluded that the success of all-ceramic restorations involves excellent esthetic results with a satisfactory integration of them to the gingival margin. However, the reproduction of the lifelike esthetic appearance of natural teeth and the visualization of the final results before definitive procedures are essential to obtain the clinical success.
